# Supplementary material for: Association between cumulative exposure periods of flupentixol or any antipsychotics and risk of lung cancer
Source: Commun Med (Lond). 2023 Sep 26;3:126. doi: 10.1038/s43856-023-00364-z (PMC10522572; doi:10.1038/s43856-023-00364-z)
Supplement: Supplementary file 2 — Supplementary Information [file 43856_2023_364_MOESM2_ESM.pdf]

Supplementary Information

Association between cumulative exposure periods of flupentixol or any antipsychotics and risk of lung cancer

Yi Chai PhD [1,2] †, Rachel Yui Ki Chu MPH [1] †, Yuqi Hu MSc [1] †, Ivan Chun Hang Lam MPharm [1], Franco Wing Tak Cheng MClinPharm [1], Hao Luo PhD [2-4], Martin Chi Sang Wong MD [5], Sandra Sau Man Chan FRCPsych(UK) [6], Esther Wai Yin Chan PhD [1,7], Ian Chi Kei Wong PhD [1,7]*, Francisco Tsz Tsun Lai PhD [1,7,8] *

† These authors contributed equally

* These authors jointly supervised this work

1 Centre for Safe Medication Practice and Research, Department of Pharmacology and Pharmacy, Li Ka Shing Faculty of Medicine, The University of Hong Kong, Hong Kong SAR, China

2 The Hong Kong Jockey Club Center for Suicide Research and Prevention, The University of Hong Kong, Hong Kong SAR, China

3 Department of Social Work and Social Administration, Faculty of Social Sciences, The University of Hong Kong, Hong Kong SAR, China

4 Sau Po Centre on Ageing, The University of Hong Kong, Hong Kong SAR, China

5 Centre for Health Education and Health Promotion, The Jockey Club School of Public Health and Primary Care, Faculty of Medicine, The Chinese University of Hong Kong, Hong Kong SAR, China

6 Department of Psychiatry, Faculty of Medicine, The Chinese University of Hong Kong, Hong Kong SAR, China

7 Laboratory of Data Discovery for Health ($D^2$4H), Hong Kong Science and Technology Park, Hong Kong SAR, China

8 Department of Family Medicine and Primary Care, Li Ka Shing Faculty of Medicine, The University of Hong Kong, Hong Kong SAR, China

Supplementary Table 1. Coding details for covariates identification

	Coding details
**Previous medications**	
NSAIDs	Ibuprofen, diclofenac, indomethacin, ketoprofen, ketorolac, mefenamic acid, naproxen, piroxicam, celecoxib, etoricoxib
Statins	Atorvastatin, fluvastatin, lovastatin, rosuvastatin, pravastatin, simvastatin
Aspirin	Aspirin
Metformin	Metformin
**Comorbidities**	
Tobacco use	ICD-9: 305.1, V15.82
Diabetes	ICD-9: 250
COPD	ICD-9: 490-496
Hypertension	ICD-9: 401
Hyperlipidemia	ICD-9: 272.4
Cirrhosis	ICD-9: 571
CKD	ICD-9: 585.9
Peptic ulcer	ICD-9: 533.0-533.7
Pneumonia	ICD-9: 486
Schizophrenia	ICD-9: 295.0-295.9
Depressive disorders	ICD-9: 296.2-296.3, 300.4, 311
Anxiety disorders	ICD-9: 300.0, 300.2, 300.3
Bipolar disorders	ICD-9: 296.0, 296.1, 296.4–296.8
Personality disorders	ICD-9: 301
Delusional disorders	ICD-9: 297
Other nonorganic psychoses	ICD-9: 298
Dementia	ICD-9: 290, 294.1, 294.2, 294.8, 331.0, 331.1, 331.82

NSAIDs: non-steroidal anti-inflammatory drugs; COPD: chronic obstructive pulmonary diseases; CKD: chronic kidney disease

Supplementary Table 2. Descriptive statistics of the study sample by exposure group for flupentixol and any antipsychotics

	Flupentixol			Any antipsychotics		
	**0-365 days**	**366-1825 days**	**1826+ days**	**1-365 days**	**366-1825 days**	**1826+ days**
**Patients, No.**	68995	952	836	37160	18781	14842
**Gender**						
Female	24853 (36.02)	334 (35.08)	256 (30.62)	1210 (32.62)	7936 (42.26)	5387 (36.30)
Male	44142 (63.98)	618 (64.92)	580 (69.38)	25040 (67.38)	10845 (57.74)	9455 (63.70)
**Age**						
18-24	0	0	0	0	0	0
25-44	1069 (1.55)	60 (6.30)	15 (1.79)	451 (1.21)	390 (2.08)	303 (2.04)
45-64	15707 (22.77)	517 (54.31)	430 (51.44)	6724 (18.09)	4112 (21.89)	5818 (39.20)
65-84	36761 (53.28)	362 (38.03)	376 (44.98)	20772 (55.90)	9446 (50.30)	7281 (49.06)
85+	15458 (22.40)	13 (13.66)	15 (1.79)	9213 (24.79)	4833 (25.73)	1440 (9.70)
Mean (SD), y	73.87 (12.73)	61.87 (10.90)	63.96 (9.28)	75.45 (12.15)	74.32 (13.40)	68.02 (11.97)
**Previous medications**						
NSAIDs	34256 (49.65)	463 (48.63)	460 (55.02)	18588 (50.02)	8879 (47.28)	7712 (51.96)
Statins	19141 (27.74)	143 (15.02)	213 (25.48)	11280 (30.36)	4587 (24.42)	3630 (24.46)
Aspirin	27489 (39.84)	139 (14.60)	131 (15.67)	16951 (45.62)	7212 (38.40)	3596 (24.23)
Metformin	13020 (18.87)	158 (16.60)	184 (22.01)	7110 (19.13)	3133 (16.68)	3119 (21.01)
**Comorbidities**						
Tobacco use	557 (0.83)	3 (0.33)	14 (1.71)	316 (0.87)	119 (0.66)	139 (0.96)
Diabetes	13501 (20.10)	123 (13.64)	146 (17.85)	7852 (21.63)	3297 (18.28)	2621 (18.03)
COPD	7139 (10.63)	44 (4.88)	47 (5.75)	4417 (12.17)	1708 (9.47)	1105 (7.60)
Hypertension	24818 (36.95)	159 (17.63)	223 (27.26)	14538 (40.17)	6568 (36.41)	4049 (27.86)
Hyperlipidemia	7301 (10.87)	49 (5.43)	75 (9.17)	4311 (11.87)	1710 (9.48)	1404 (9.66)
Cirrhosis	1205 (1.79)	14 (1.55)	22 (2.69)	728 (2.01）	270 (1.50)	243 (1.67)
CKD	1956 (2.91)	8 (0.89)	5 (0.61)	1506 (4.15)	288 (1.60)	175 (1.20)
Peptic ulcer	457 (0.68)	1 (0.11)	4 (0.49)	269 (0.74)	114 (0.63)	79 (0.54)
Pneumonia	14512 (21.61)	85 (9.42)	98 (11.98)	8366 (23.04)	3904 (21.64)	2425 (16.69)
Schizophrenia	12071 (17.97)	666 (73.84)	703 (85.94)	1355 (3.73)	3458 (19.17)	8627 (59.36)
Depressive disorders	9070 (13.51)	134 (14.86)	78 (9.54)	4079 (11.24)	3132 (17.36)	2071 (14.25)
Anxiety disorders	2321 (3.46)	27 (2.99)	13 (1.59)	1075 (2.96)	757 (4.20)	529 (3.64)
Bipolar disorders	2061 (3.07)	39 (4.32)	22 (2.69)	514 (1.42)	621 (3.44)	987 (6.80)
Personality disorders	799 (1.19)	25 (2.77)	11 (1.34)	255 (0.70)	249 (1.38)	331 (2.28)
Delusional disorders	3038 (4.52)	78 (8.65)	50 (6.11)	866 (2.39)	1186 (6.57)	1114 (7.66)
Other nonorganic psychoses	5383 (8.02)	130 (14.41)	99 (12.10)	2291 (6.31)	1611 (8.93)	1710 (11.77)
Dementia	19245 (28.66)	44 (4.88)	35 (4.28)	9483 (26.12)	7783 (43.15)	2058 (14.16)

NSAIDs: non-steroidal anti-inflammatory drugs; COPD: chronic obstructive pulmonary diseases; CKD: chronic kidney disease

Supplementary Table 3. Full results of covariates from the main analysis

	Total sample		Males		Females	
	**Analysis One**	**Analysis Two**	**Analysis One**	**Analysis Two**	**Analysis One**	**Analysis Two**
**Cumulative use of other antipsychotics***						
366-1825 days	0.42 (0.39-0.46)	-	0.41 (0.37-0.46)	-	0.43 (0.39-0.49)	-
1826+ days	0.56 (0.50-0.61)	-	0.54 (0.48-0.62)	-	0.58 (0.50-0.67)	-
**Previous medications**						
NSAIDs	1.33 (1.26-1.41)	1.33 (1.26-1.41)	1.34 (1.25-1.44)	1.34 (1.25-1.44)	1.32 (1.20-1.45)	1.31 (1.20-1.45)
Statins	1.02 (0.94-1.10)	1.02 (0.94-1.10)	1.04 (0.94-1.15)	1.04 (0.94-1.15)	0.99 (0.88-1.13)	1.00 (0.88-1.13)
Aspirin	0.77 (0.72-0.82)	0.77 (0.72-0.82)	0.77 (0.71-0.83)	0.77 (0.71-0.83)	0.77 (0.69-0.86)	0.77 (0.69-0.86)
Metformin	0.95 (0.85-1.05)	0.95 (0.85-1.05)	0.95 (0.83-1.08)	0.95 (0.83-1.08)	0.95 (0.80-1.12)	0.95 (0.80-1.12)
**Comorbidities**						
Tobacco use	1.17 (0.90-1.53)	1.17 (0.90-1.53)	1.12 (0.84-1.49)	1.12 (0.84-1.49)	1.75 (0.89-3.45)	1.78 (0.90-3.50)
Diabetes	0.89 (0.81-0.99)	0.89 (0.81-0.99)	0.91 (0.80-1.03)	0.91 (0.80-1.03)	0.86 (0.93-1.02)	0.86 (0.93-1.02)
COPD	1.58 (1.47-1.71)	1.58 (1.47-1.71)	1.71 (1.56-1.87)	1.71 (1.56-1.87)	1.34 (1.16-1.54)	1.33 (1.16-1.54)
Hypertension	0.86 (0.81-0.92)	0.86 (0.81-0.92)	0.77 (0.71-0.84)	0.77 (0.71-0.84)	1.04 (0.94-1.16)	1.04 (0.93-1.16)
Hyperlipidemia	1.12 (1.01-1.24)	1.12 (1.02-1.24)	1.14 (1.00-1.29)	1.14 (1.00-1.29)	1.10 (0.93-1.29)	1.10 (0.93-1.29)
Cirrhosis	0.90 (0.74-1.10)	0.90 (0.74-1.10)	0.98 (0.78-1.22)	0.98 (0.78-1.22)	0.72 (0.48-1.09)	0.72 (0.48-1.09)
CKD	0.64 (0.54-0.76)	0.64 (0.54-0.76)	0.63 (0.52-0.78)	0.63 (0.52-0.78)	0.66 (0.48-0.90)	0.66 (0.48-0.90)
Peptic ulcer	0.96 (0.70-1.33)	0.96 (0.70-1.33)	0.70 (0.46-1.06)	0.70 (0.46-1.06)	1.84 (1.10-3.09)	1.84 (1.10-3.09)
Pneumonia	2.36 (2.22-2.52)	2.36 (2.22-2.52)	2.27 (2.10-2.45)	2.27 (2.10-2.45)	2.57 (2.30-2.88)	2.57 (2.30-2.87)
Schizophrenia	0.47 (0.43-0.53)	0.47 (0.43-0.53)	0.48 (0.42-0.55)	0.48 (0.42-0.55)	0.48 (0.40-0.57)	0.47 (0.40-0.56)
Depressive disorders	0.61 (0.56-0.67)	0.61 (0.56-0.67)	0.60 (0.53-0.68)	0.60 (0.53-0.68)	0.63 (0.55-0.72)	0.63 (0.55-0.72)
Anxiety disorders	0.59 (0.50-0.70)	0.59 (0.50-0.70)	0.51 (0.39-0.67)	0.51 (0.39-0.67)	0.66 (0.52-0.83)	0.66 (0.52-0.83)
Bipolar disorders	0.47 (0.38-0.58)	0.47 (0.38-0.58)	0.49 (0.37-0.65)	0.49 (0.37-0.65)	0.44 (0.32-0.62)	0.44 (0.32-0.62)
Personality disorders	0.74 (0.55-0.99)	0.74 (0.55-0.99)	0.72 (0.49-1.05)	0.72 (0.49-1.05)	0.78 (0.50-1.21)	0.77 (0.50-1.21)
Delusional disorders	0.53 (0.45-0.63)	0.53 (0.45-0.63)	0.48 (0.38-0.61)	0.48 (0.38-0.61)	0.61 (0.48-0.76)	0.61 (0.48-0.76)
Other nonorganic psychoses	0.96 (0.86-1.06)	0.96 (0.86-1.06)	0.94 (0.82-1.08)	0.94 (0.82-1.08)	0.98 (0.83-1.17)	0.98 (0.83-1.16)
Dementia	0.45 (0.42-0.49)	0.45 (0.42-0.49)	0.45 (0.41-0.50)	0.45 (0.41-0.50)	0.45 (0.40-0.50)	0.45 (0.40-0.50)

Analysis one is to investigate the association between the lung cancer and different cumulative exposure time of flupentixol, adjusting for other antipsychotics and all interested covariates.

Analysis two is to investigate the association between the lung cancer and different cumulative exposure time of all antipsychotics (including flupentixol), adjusting for all interested covariates.

NSAIDs: non-steroidal anti-inflammatory drugs; COPD: chronic obstructive pulmonary diseases; CKD: chronic kidney disease

* Reference group is 0-365 days

Supplementary Table 4. Results from the sensitivity analysis of excluding patients with previous exposure to drugs which were associated with lung cancer risk reduction

	Total sample			Males			Females		
	Cases, No. (%)	Controls, No. (%)	OR (95% CI)	Cases, No. (%)	Controls, No. (%)	OR (95% CI)	Cases, No. (%)	Controls, No. (%)	OR (95% CI)
**Patients, No.**	1410	4000		1004	2952		406	1048	
**Analysis one**									
**Cumulative use of flupentixol**									
0-365 days	1393 (98.79)	3885 (97.13)	ref	998 (99.40)	2861 (96.92)	ref	395 (97.29)	1024 (97.71)	ref
366-1825 days	10 (0.71)	53 (1.33)	0.69 (0.33-1.47)	5 (0.50)	41 (1.39)	0.60 (0.22-1.62)	5 (1.23)	12 (1.15)	1.07 (0.34-3.43)
1826+ days	7 (0.50)	62 (1.55)	0.37 (0.16-0.84)	1 (0.01)	50 (1.69)	0.07 (0.009-0.52)	6 (1.48)	12 (1.15)	1.62 (0.55-4.77)
**Analysis two**									
**Cumulative use of any antipsychotics**									
1-365 days	1141 (80.92)	2676 (66.90)	ref	874 (87.05)	2160 (73.17)	ref	281 (69.21)	604 (57.63)	ref
366-1825 days	172 (12.20)	868 (21.70)	0.53 (0.43-0.65)	85 (8.47)	532 (18.02)	0.51 (0.39-0.67)	80 (19.70)	307 (29.29)	0.60 (0.44-0.83)
1826+ days	97 (6.88)	456 (11.40)	0.62 (0.47-0.81)	45 (4.48)	260 (8.81)	0.49 (0.34-0.71)	45 (11.08)	137 (13.07)	0.86 (0.57-1.31)

Analysis one is to investigate the association between the lung cancer and different cumulative exposure time of flupentixol, adjusting for other antipsychotics and all interested covariates.
Analysis two is to investigate the association between the lung cancer and different cumulative exposure time of all antipsychotics (including flupentixol), adjusting for all interested covariates.

Supplementary Table 5. Results from the sensitivity analysis in which each patient can be selected as a control for an unlimited number of cases

	Total sample			Males			Females		
	**Cases, No. (%)**	**Controls, No. (%)**	**OR (95% CI)**	**Cases, No. (%)**	**Controls, No. (%)**	**OR (95% CI)**	**Cases, No. (%)**	**Controls, No. (%)**	**OR (95% CI)**
**Patients, No.**	1410	4000		1004	2952		406	1048	
**Analysis one**									
**Cumulative use of flupentixol**									
0-365 days	6361 (98.85)	62613 (97.30)	ref	4075 (98.86)	40079 (97.24)	ref	2286 (98.83)	22534 (97.42)	ref
366-1825 days	38 (0.59)	930 (1.45)	0.63 (0.45-0.88)	26 (0.63)	596 (1.45)	0.76 (0.51-1.14)	12 (0.52)	334 (1.44)	0.45 (0.25-0.82)
1826+ days	36 (0.56)	805 (1.25)	0.72 (0.51-1.02)	21 (0.51)	543 (1.32)	0.69 (0.44-1.08)	15 (0.65)	262 (1.13)	0.78 (0.46-1.35)
**Analysis two**									
**Cumulative use of any antipsychotics**									
1-365 days	4793 (74.48)	32216 (50.07)	ref	3204 (77.73)	21892 (53.11)	ref	1589 (68.70)	10549 (45.61)	ref
366-1825 days	845 (13.13)	17754 (27.59)	0.42 (0.39-0.46)	445 (10.80)	10220 (24.79)	0.41 (0.36-0.45)	400 (17.29)	7509 (32.46)	0.44 (0.39-0.50)
1826+ days	797 (12.39)	14378 (22.34)	0.52 (0.47-0.57)	473 (11.48)	9106 (22.09)	0.49 (0.43-0.55)	324 (14.01)	5072 (21.93)	0.57 (0.49-0.66)

Analysis one is to investigate the association between the lung cancer and different cumulative exposure time of flupentixol, adjusting for other antipsychotics and all interested covariates.
Analysis two is to investigate the association between the lung cancer and different cumulative exposure time of all antipsychotics (including flupentixol), adjusting for all interested covariates.
