# Supplementary material for: Association between cumulative exposure periods of flupentixol or any antipsychotics and risk of lung cancer
Source: Commun Med (Lond). 2023 Sep 26;3:126. doi: 10.1038/s43856-023-00364-z (PMC10522572; doi:10.1038/s43856-023-00364-z)
Supplement: Supplementary file 3 — Reporting summary [file 43856_2023_364_MOESM3_ESM.pdf]

nature portfolio

Corresponding author(s): Francisco Lai

Last updated by author(s): Aug 11, 2023

Reporting Summary

Nature Portfolio wishes to improve the reproducibility of the work that we publish. This form provides structure for consistency and transparency in reporting. For further information on Nature Portfolio policies, see our Editorial Policies and the Editorial Policy Checklist.

Statistics

For all statistical analyses, confirm that the following items are present in the figure legend, table legend, main text, or Methods section.

n/a	Confirmed	
☐	☒	The exact sample size (*n*) for each experimental group/condition, given as a discrete number and unit of measurement
☐	☒	A statement on whether measurements were taken from distinct samples or whether the same sample was measured repeatedly
☐	☒	The statistical test(s) used AND whether they are one- or two-sided<br>*Only common tests should be described solely by name; describe more complex techniques in the Methods section.*
☐	☒	A description of all covariates tested
☐	☒	A description of any assumptions or corrections, such as tests of normality and adjustment for multiple comparisons
☐	☒	A full description of the statistical parameters including central tendency (e.g. means) or other basic estimates (e.g. regression coefficient) AND variation (e.g. standard deviation) or associated estimates of uncertainty (e.g. confidence intervals)
☐	☒	For null hypothesis testing, the test statistic (e.g. *F*, *t*, *r*) with confidence intervals, effect sizes, degrees of freedom and *P* value noted<br>*Give P values as exact values whenever suitable.*
☒	☐	For Bayesian analysis, information on the choice of priors and Markov chain Monte Carlo settings
☒	☐	For hierarchical and complex designs, identification of the appropriate level for tests and full reporting of outcomes
☒	☐	Estimates of effect sizes (e.g. Cohen's *d*, Pearson's *r*), indicating how they were calculated

Our web collection on statistics for biologists contains articles on many of the points above.

Software and code

Policy information about availability of computer code

Data collection	All electronic health records were downloaded from the Hong Kong Clinical Data Analysis and Reporting System (CDARS) managed by the Hong Kong Hospital Authority
Data analysis	The statistical software R (version 4.1.2) was used for all analyses. The code used for this study is available on Zenodo (DOI: 10.5281/zenodo.8009776)

For manuscripts utilizing custom algorithms or software that are central to the research but not yet described in published literature, software must be made available to editors and reviewers. We strongly encourage code deposition in a community repository (e.g. GitHub). See the Nature Portfolio guidelines for submitting code & software for further information.

Data

Policy information about availability of data

All manuscripts must include a data availability statement. This statement should provide the following information, where applicable:

- Accession codes, unique identifiers, or web links for publicly available datasets
- A description of any restrictions on data availability
- For clinical datasets or third party data, please ensure that the statement adheres to our policy

Data are not available as the data custodians (the Hospital Authority and the Department of Health of Hong Kong SAR) have not given permission for sharing due to patient confidentiality and privacy concerns. Local academic institutions, government departments, or non-governmental organizations may apply for the access to

data through the Hospital Authority's data sharing portal (https://www3.ha.org.hk/data). The numerical data underlying Figure 2 were shown in tables in Figure 2. All other data are available from the corresponding author (or other sources, as applicable) on reasonable request.

Research involving human participants, their data, or biological material

Policy information about studies with human participants or human data. See also policy information about sex, gender (identity/presentation), and sexual orientation and race, ethnicity and racism.

Reporting on sex and gender	The term "sex" was used throughout the whole manuscript. The sex was considered in the study design. The sex of participants was determined based on self-report when participants showed in the health care system. Sex-stratified analysis was conducted. A total of 70783 people were included in the analysis, including 45340 males and 25443 females.
Reporting on race, ethnicity, or other socially relevant groupings	The current study is based on the electronic health records and no socially relevant categorization variables were included.
Population characteristics	See above.
Recruitment	All electronic health recorded were directly downloaded from the Hong Kong Clinical Data Analysis and Reporting System (CDARS)
Ethics oversight	Institutional Review Board of the University of Hong Kong/ Hospital Authority Hong Kong West Cluster

Note that full information on the approval of the study protocol must also be provided in the manuscript.

Field-specific reporting

Please select the one below that is the best fit for your research. If you are not sure, read the appropriate sections before making your selection.

☐ Life sciences ☒ Behavioural & social sciences ☐ Ecological, evolutionary & environmental sciences

For a reference copy of the document with all sections, see nature.com/documents/nr-reporting-summary-flat.pdf

Behavioural & social sciences study design

All studies must disclose on these points even when the disclosure is negative.

Study description	A quantitative case-control study
Research sample	All patients who received prescriptions of any antipsychotics between January 01, 2001, and August 31, 2022 in the Hong Kong Clinical Data Analysis and Reporting System (CDARS). CDARS is a territory-wide database developed by the Hospital Authority (HA), a statutory body managing all public hospitals and providing healthcare services for more than 7 million Hong Kong residents.
Sampling strategy	No sample-size calculation was performed since this study used the huge volumes of territory-wide electronic health records to do the analysis. The minimum sample size calculation for traditional hypothesis testing does not apply.
Data collection	All electronic health recorded were directly downloaded from the Hong Kong Clinical Data Analysis and Reporting System (CDARS) using computers.
Timing	From September 1, 2022, to October 31, 2022
Data exclusions	Patients who 1) had missing values on age, sex, and date of death (N=239); 2) had incorrect records (i.e., the death date was before the date of first prescription of antipsychotics) (N=49); 3) had a diagnosis of lung cancer before or at the date of index prescription of antipsychotics (N=17018); or 4) had a diagnosis of lung cancer before January 01, 2003 (N=663), were excluded.
Non-participation	No participants dropped out/declined participation.
Randomization	Participants were not allocated into experimental groups.

Reporting for specific materials, systems and methods

We require information from authors about some types of materials, experimental systems and methods used in many studies. Here, indicate whether each material, system or method listed is relevant to your study. If you are not sure if a list item applies to your research, read the appropriate section before selecting a response.

Materials & experimental systems

n/a	Involved in the study
☒	☐ Antibodies
☒	☐ Eukaryotic cell lines
☒	☐ Palaeontology and archaeology
☒	☐ Animals and other organisms
☒	☐ Clinical data
☒	☐ Dual use research of concern
☒	☐ Plants

Methods

n/a	Involved in the study
☒	☐ ChIP-seq
☒	☐ Flow cytometry
☒	☐ MRI-based neuroimaging
